# The Value of Data: Applying a Public Value Model to the English National Health Service

**DOI:** 10.2196/15816

**Published:** 2020-03-27

**Authors:** James Wilson, Daniel Herron, Parashkev Nachev, Nick McNally, Bryan Williams, Geraint Rees

**Affiliations:** 1 Department of Philosophy University College London London United Kingdom; 2 Research & Development UCL Hospitals/UCL Biomedical Research Centre London United Kingdom; 3 Institute of Neurology University College London London United Kingdom; 4 UCL Institute of Cardiovascular Sciences University College London London United Kingdom; 5 Institute of Cognitive Neuroscience University College London London United Kingdom; 6 Faculty of Life Sciences University College London London United Kingdom; 7 Wellcome Trust Centre for Neuroimaging University College London London United Kingdom

**Keywords:** health policy, innovation, public value, intellectual property, NHS Constitution

## Abstract

Research and innovation in biomedicine and health care increasingly depend on electronic data. The emergence of data-driven technologies and associated digital transformations has focused attention on the value of such data. Despite the broad consensus of the value of health data, there is less consensus on the basis for that value; thus, the nature and extent of health data value remain unclear. Much of the existing literature presupposes that the value of data is to be understood primarily in financial terms, and assumes that a single financial value can be assigned. We here argue that the value of a dataset is instead relational; that is, the value depends on who wants to use it and for what purposes. Moreover, data are valued for both nonfinancial and financial reasons. Thus, it may be more accurate to discuss the values (plural) of a dataset rather than the singular value. This plurality of values opens up an important set of questions about how health data should be valued for the purposes of public policy. We argue that public value models provide a useful approach in this regard. According to public value theory, public value is created, or captured, to the extent that public sector institutions further their democratically established goals, and their impact on improving the lives of citizens. This article outlines how adopting such an approach might be operationalized within existing health care systems such as the English National Health Service, with particular focus on actionable conclusions.

## Background

Research and innovation in biomedicine and health care increasingly depend on electronic data. Health care systems are data-rich environments, within which data are collected, used, and shared. The emergence of data-driven technologies and associated digital transformations has brought the value of such data into focus; in particular, the “value proposition” focuses on better patient outcomes relative to cost [1,2]. Despite the broad consensus on the value of data, the basis for that value, and thus its nature and extent, is not clear. It is essential that the conceptual framework within which any valuation of data can be coherently couched is clarified. We here argue that the idea of public value holds much promise for elucidating the value of public sector data. We further highlight how such an approach might be operationalized within health care systems such as England's National Health Service (NHS), with particular focus on reaching actionable conclusions.

## Defining Value

To clarify the value of data, it is useful to begin by focusing on the elements of *data* and *value* as separate entities*.* Most familiar everyday goods such as cars and tables are rival goods: they can only be in one place at a time, and may only be possessed or used by a single user. Moreover, many rival goods in health care research and innovation (eg, tissue or DNA samples) are nondurable such that consumption destroys the good and allows only one user to enjoy it. In contrast, data are a nonrival and durable good: the same data items can simultaneously be used (and subsequently copied for reuse) by many different agents for multifarious purposes without difficulty. For example, the same underlying data can be used to provide direct care to patients, help plan future services, conduct multiple health care research projects, and realize economic benefits through commercialization. Although datasets themselves are nonrival in use and are durable, these same properties may not apply to their value [3]. Data that provide a competitive advantage will lose much of their value or usefulness if they become common knowledge. Data used for location-sensitive advertising while an individual is in a particular shop will quickly depreciate in value. Thus, the fact that data *can* be used for an infinite variety of purposes simultaneously does not necessarily mean that they *will* be, or even that they should be.

There are several ways in which access to goods that are intrinsically nonrival can be governed for reasons of social policy. Commonly used models conceive of data as: (1) a private good, in which a dataset that could be shared much more widely is not, perhaps for ethical or legal reasons, or due to a competitive advantage (as in test data exclusivity in clinical trials); (2) a licensed good, in which access to the data is controlled and managed via approval processes that stipulate factors such as the purposes for which the data can be used; or (3) an open commons, in which the data are openly accessible for anyone to reuse or add value [4]. The optimal governance solution for data in each case will require balancing the ability to generate the data, the opportunity to make use of it, and maintaining the underpinning social license.

The starting point for considering value within the framework of public policy is often economic or market value. This may be driven by an assumption that cost-benefit analysis is the best way to approach public sector decision making, but may also reflect the perceived importance of commercial imperatives within public sector data policy. In any case, this framing naturally excludes valuations for which there is no market, and degrades those where commodification is difficult or impossible. In addition, some aspects of value can be difficult to assess financially. For example, innovation in health care technology from data can create intangible assets that are not physical in nature, such as patents, copyrights, software, goodwill, and brand recognition. Although such intangibles are frequently difficult to value, they nevertheless are considered to represent a significant proportion of the value that can be created from data [5].

It is important to consider values other than financial value as relevant to health care. For example, patients value being treated with respect by health care professionals, and also value being told candidly when something has gone wrong in their care or treatment, but it would be a mistake to consider either of these values as primarily financial. In the context of health care, nonfinancial values are often grouped together under the heading of social value judgments [6].

The value of datasets should be understood in relational terms. That is, the value is not intrinsic to the dataset itself, but rather depends on the varied purposes for which it will be used. Different agents may value the same dataset for different reasons and to different extents. Access to vast datasets of patient-level data will be mission-critical for a company whose main product is health data analytics, but will be merely useful and nonessential for a company with a different focus. Moreover, the same agent may simultaneously value a given dataset in multiple ways. For example, patients may simultaneously place a nonfinancial value on their health data—insofar as they value their privacy, and the assurance of confidentiality within clinical encounters—and a financial value, insofar as they might be willing to sell rights to use the data to researchers or commercial companies. In summary, it may be less misleading to discuss the *values* (plural) of a dataset than to imply that there is a singular value of a dataset, regardless of the context.

The financial value of particular datasets will be determined by a combination of supply-side and demand-side features. On the supply side, these features will include: (1) data quality, (2) the format in which the data are stored and the extent to which this format is machine-readable, (3) the ability to link the data, (4) the type of data (eg, identifiable, anonymized, or aggregate), (5) the quantity of data, and (6) the degree to which the data are actionable [7]. On the demand side, the main factors determining the value will include: (1) the use of the data determined by an organization, and the amount of data required to fulfill this purpose; (2) the wealth and willingness to pay of the organization; and (3) the relative cost of obtaining access to data elsewhere [7]. While much attention is rightly devoted to large-scale datasets that could be viewed as strategic assets, questions about the value of data also need to be addressed within locally implemented systems. Textbox 1 provides a worked example of the value of data in an appointment scheduling system.

**The value of data in an artificial intelligence appointment scheduling system.**

An innovative artificial intelligence (AI) solution to health care challenges such as optimizing hospital nonattendance [8] illustrates the intricacies of valuing data. The value of reducing nonattendance is two-fold: first, it accelerates the delivery of care for patients, achieving better outcomes faster; and second, it enhances the productivity of the institution, producing a greater health care impact for the quantity of dedicated resources. The problem is too complex to be solved intuitively*,* and therefore requires a solution drawn from historical data, such as a predictive model of nonattendance that enables appropriately focused reminding. The higher the predictive fidelity, the more efficient the interventions introduced will be to maximize attendance.

The extent to which a datum contributes to the fidelity of the model will depend on the complexity of the underlying problem: simple problems are data-efficient, requiring only small samples, whereas complex problems such as the present example are often profligate, requiring large, diverse collections of data.

Although the marginal value of a single datum might be considered low, the value of a relatively small increase in predictive performance can be very high. A large hospital trust could easily have around 1 million scheduled events a year; thus, savings of even a few pence per appointment could be cumulatively substantial. Moreover, the social value of even a single instance of death or major disability thereby prevented is obviously far greater than the cost of computational modeling and the increasingly inexpensive digital systems required for its implementation. Accordingly, the marginal contribution of a datum needs to be contextualized by its ultimate impact.

Could we nonetheless assign a value to the data before the modeling work has been done? The answer in this case is clearly no, given that the complexity of the problem is only revealed in the course of analysis once data of the necessary scale have been evaluated. Just as the statistical power of complex models cannot be estimated from small, pilot samples, the maximum achievable predictive performance (ie, the determinant of value in this case) can only be known once the modeling work is complete. Reflection on the multifactorial nature of hospital attendance could allow one to *guess* that models of dimensionality only supported by large-scale data would be optimal, but this can only be *confirmed* in a post hoc context.

Even if the model is demonstrated to be stable over time at the source institution, generalizability to other institutions cannot be assumed. Indeed, a more sophisticated model is likely to be closely tailored to the local population. Therefore, the features of a model that enhance its *local* value may degrade its *global* value beyond the confines of the specific institution. This is not a reason to keep models simple, but rather highlights the need for building a distributed machine-learning infrastructure that allows individual institutions to build their own models rather than relying on generic models drawn from pooled data.

## The Financial Value of Data

There is a widespread assumption that the value of public sector data is not being realized to its full capacity, and that this value needs to be optimized [2,9]. Nonetheless, how to measure the value of data for the purposes of public policy is uncertain. It is therefore unclear what a commitment to optimize this value might mean.

From an economic perspective, it might initially be tempting to think that the correct value of data is its price within a market, which will be determined by the operation of supply and demand. Optimizing the value of public sector health data might then be equated with maximizing the resale value of the data. However, such an approach is too narrowly focused on economic value, and completely disregards some of the nonfinancial ways in which data are valued.

Even in narrow financial terms, there will be circumstances in which making the data available for free or on a cost-recovery basis will be a more successful strategy, if doing so stimulates markets to build services on top of the data, which then create employment and tax revenue. For example, the US government’s investment in the Global Positioning System, which it makes freely available, created new private markets by adding value to smartphones and to satellite navigation systems, which far outweighs the cost of the initial investment [10].

Thus, there is no reason to think that maximizing the amount that a business pays for access to public sector health data would also maximize the amount of economic value created. Moreover, asymmetric information might dominate health care markets for data, by which the commercial parties involved in such transactions are likely to possess much greater material knowledge of the potential uses of health care data and the likely future products. This could lead to suboptimal pricing of the data, moral hazard, or poor selection of market opportunities.

Governments should be primarily concerned with the economic value created through the use of the data over a life cycle rather than what could be gained from its initial sale. The success of data repositories such as the European Nucleotide Archive [11] illustrates this point vividly, as the biological sequences are made available to researchers free of charge, thereby vastly increasing the efficiency of related research. There is a consensus within the omics community that such curation and repositories are effective ways of creating value, despite (and partly because of) the fact that the sequences are given away free of charge.

Working out the optimal price and commercial strategies for public sector health care data will be complex, even in narrow financial terms. There will rarely be a matter of a single monolithic entity that controls all of the public sector health data in a particular state; rather, there is likely to be fragmentation at a regional or functional basis. This greatly complicates the economic picture. First, in cases with multiple potential sources for data—for example, if a company requires a clinical dataset from 1 million patients to build a tool, but could obtain a dataset from distinct regional bodies—competition between these bodies could significantly reduce the market price, leading to a less than optimal capture of the value by the public sector. This is one reason why the UK government’s Office for Life Sciences has proposed that any commercial arrangements made by local NHS organizations should not be allowed to “undermine, inhibit or impact” the ability of the system at a national level to maximize the value of NHS data [12].

Second, to the extent that access to health data is commercialized, there is room for disagreement about where the money should go (ie, individual patients, hospitals, regional bodies, or the health care system as a whole) and the extent to which this income should be used for delivery of care or to further enhance the system’s ability to commercialize data. Third, there may be no guarantee that the value created through the release of a particular dataset for free or on a cost-recovery basis can be captured within its territory of origin. Even if these issues can be addressed, the data under control of health care systems will be derived from both the patients and from the operation of the health care system and its employees. Therefore, determining the ultimate recipient of reimbursement for any value remains to be resolved.

## Public Value

Any policy that aims to optimize the value of public sector health data also needs to give due weight to the nonfinancial values that health systems are seeking to promote and protect. Health systems most obviously aim to restore those who are sick back to normal functioning, and to prevent the onset of disease. However, they also embed other values such as confidentiality and respect for autonomy. These values can be realized through role-based access controls that ensure access to patient data is only on a need-to-know basis, and in the ways in which health systems allow patients to choose how their identifiable health data will be shared. These values of confidentiality and respect for autonomy may come into potential conflict with the financial value to be realized through the commercialization of data; thus, any attempt to optimize value will need to take this into account in such cases.

In brief, the kind of value that public sector organizations should be seeking to optimize is not in any straightforward sense a financial value. Because of the interplay of financial and nonfinancial values, concepts such as “value chain,” “adding value,” and “value proposition” need to be translated, and partially transformed, from a business context. The public management theorist Mark Moore labels the kind of value that the public sector should be aiming to create as public value [13]. Public value is created, or captured, to the extent that public sector institutions further their democratically established goals or improve the lives of citizens [14].

Discussion of the factors contributing to enhancement of public value thus requires clarity about the purposes of different parts of the public sector, and how they fit together into an overall conception of the public good. Although realizing this type of clarity will require the public articulation and discussion of value judgments, this is a virtue rather than a vice in any account of value that will guide public policy.

In some cases, a public sector organization will have already articulated a set of values or principles that together express what is needed for the institution to do its job well. England's NHS offers a good example of such an organization. The constitution of the NHS establishes seven core principles: (1) the aim is to provide a comprehensive service, available to all; (2) access to those services will be based on clinical need not ability to pay; (3) care should be delivered with the highest standards of excellence and professionalism; (4) care should be patient-centered; (5) the NHS should be integrated across organizational boundaries; (6) the NHS should be committed to providing good value for tax payers’ money; and (7) the NHS should be accountable to the public, communities, and patients [15].

These principles could be used to help specify a model of public value for the NHS, which could inform questions about the value of data. For example, public value would be created if investing in interoperability and linkage of datasets allows for a system of care that is better integrated across organizational boundaries (principle 5). Similarly, implementing changes that improve value for money would create public value (principle 6).

## Crowding Out, Perverse Incentives, and the Optimization of Public Value

There are good reasons for considering that, all else being equal, reducing the costs of a service to citizens while maintaining its quality will increase public value, even in public sector organizations that are not as explicit as the NHS in adopting this as a core principle. However, some or many citizens will not accept that the importance of value for money provides a reason to commercialize access to goods that were previously either not available or available on a noncommercial basis. The introduction of commercial motives may be resisted for many reasons, including that it may crowd out altruistic or solidaristic motivations [16,17]. Other citizens may worry that such commercialization could undermine the system’s core focus on meeting patient needs: in a system that is chronically short of money, managers are likely to take an opportunity to make extra money to cover shortfalls. Over time, the system may change so that rather than being perceived as extra cash, this resource would become part of the core budget. The system could then develop incentives to improve its ability to sell access to the data through employment of skilled professionals.

To the extent that such concerns are widespread—and a public sector organization is committed to a principle similar to the NHS’s seventh principle of accountability to the public, communities, and patients—they will need to be taken seriously. Citizens’ reasonable expectations about how the system should (and does) operate are an important force shaping public value, not least because of the need to maintain a social license [18,19]. These debates will require particular nuance and sensitivity in cases where—as perhaps in the NHS—there is a significant gap between the reality of the commercially funded and supplied nature of pharmaceuticals and medical devices, and the picture that some citizens have of a proudly noncommercial system.

However, completely avoiding commercial entanglements would cause conflict with other aspects of public value. The public sector will rarely have the skills to optimally innovate on its own, and to insist on complete lack of commercial involvement would result in delivering suboptimal care. Of course, the other extreme possibility—adopting a blanket policy of allowing commercial access to data without charge—would be in conflict with further aspects of public value. Although there will be circumstances in which making data available without charge to commercial companies will be reasonable from the perspective of public value, if those data will be used to design devices or services that will then be sold back to the public sector, there is room for concern about the fairness of the exchange.

## Conclusion

If implemented without adequate thought, the attempt to realize the financial value of public sector health data through commercialization could destabilize the broader nonfinancial values that public sector health organizations aim to promote. Introducing the idea of public value provides a more clearly expressed overview of the challenge governments face in optimizing the value of health data, but cannot by itself resolve conflicts within competing conceptions of the public good.

Detailed discussion of what a successful public value account will look like is beyond the scope of this article; however, it is important to note some key elements that will be shared by all such accounts. First, public value presupposes a background public political culture, which shapes the conventions within which democratic deliberation takes place [20]. Political cultures, and their animating values, typically shift much more slowly than specific policy choices. The core elements of the public culture of most Western societies that profoundly shape the conceptions of public value include transparency, democratic accountability, due process and the rule of law, human dignity, using resources efficiently, and maintaining public trust.

Second, public value approaches require domain-specific goals and values for different elements of public services, which specify the requirements for the service to do its job well. Here, we have provided examples of the NHS constitution and the AI appointment scheduling system that represent these values at a high level and at a more specific level, respectively. These goals and values gain their legitimacy from their congruence with the background values that structure the public political culture, the agreed direction of government policy, and the results of public deliberation. In cases requiring changes to the balance or specification of domain-specific values (eg, rapidly shifting possibilities as a result of the opportunities created by AI), public deliberation will be needed within the framework set by the background values on how best to specify the approach taken for a particular domain or institution. Public value approaches have been worked out in more detail in some cases outside of the realm of data policy, such as for priority setting in health care [21,22].

Constructing an adequate model of public value for public sector data will require both articulation of the goals and values implicit in different public services, and open public dialogue about how best to specify these goals and values in light of fast-changing circumstances. It is important to place a range of social values on the table to best understand different publics’ attitudes to public sector organizations, along with their perceived opportunities and threats, toward realizing the financial value of health data. Facilitating such conversations in a constructive manner will depend on gaining public trust. Importantly, the limits of what policy makers can do to capture the value of public sector health data while maintaining public trust cannot be determined a priori.

## Abbreviations

AI: artificial intelligence
NHS: National Health Service

## References

[1] Department of Health and Social Care (2019). Code of conduct for data-driven health and care technology.

[2] Her Majesty's Treasury (2018). The economic value of data: discussion paper.

[3] Prainsack B, Krutzinna J, Floridi L (2019). Data Donation: How to Resist the iLeviathan. The Ethics of Medical Data Donation.

[4] Benkler Y (2013). Commons and Growth: The Essential Role of Open Commons in Market Economies. The University of Chicago Law Review.

[5] Haskel J, Westlake S (2017). Capitalism Without Capital: The Rise Of The Intangible Economy.

[6] National Institute for Health and Care Excellence (2008). Social Value Judgements: Principles for the development of NICE guidance.

[7] Harwich E, Lasko-Skinnner R (2018). Making NHS data work for everyone.

[8] Nelson A, Herron D, Rees G, Nachev P (2019). Predicting scheduled hospital attendance with artificial intelligence. NPJ Digit Med.

[9] Her Majesty's Treasury (2018). Getting smart about intellectual property and other intangibles in the public sector: Budget 2018.

[10] Mazzucato M (2015). The Entrepreneurial State: Debunking Public Vs. Private Sector Myths.

[11] European Nucleotide Archive European Nucleotide Archive Browser.

[12] Department of Health & Social Care (2019). Creating the right framework to realise the benefits for patients and the NHS where data underpins innovation.

[13] Moore MH (1995). Creating Public Value: Strategic Management In Government.

[14] Moore MH (2014). Public Value Accounting: Establishing the Philosophical Basis. Public Admin Rev.

[15] Department of Health & Social Care (2015). The NHS Constitution for England.

[16] Frey B, Oberholzer-Gee F (1997). The cost of price incentives: An empirical analysis of motivation crowding-out. Am Econ Rev.

[17] Sandel M (2013). What Money Can't Buy: The Moral Limits Of Markets.

[18] Carter P, Laurie GT, Dixon-Woods M (2015). The social licence for research: why care.data ran into trouble. J Med Ethics.

[19] Taylor M, Wilson J (2019). Reasonable Expectations of Privacy and Disclosure of Health Data. Med Law Rev.

[20] Rawls J (2005). Political Liberalism.

[21] Wilson J (2016). Public Value, Maximization and Health Policy: An Examination of Hausman’s Restricted Consequentialism. Public Health Ethics.

[22] Rumbold B, Weale A, Rid A, Wilson J, Littlejohns P (2017). Public Reasoning and Health-Care Priority Setting: The Case of NICE. Kennedy Inst Ethics J.

